# Supplementary material for: Anti-Neuroinflammation Effect of Standardized Ethanol Extract of Leaves of Perilla frutescens var. acuta on Aβ-Induced Alzheimer’s Disease-like Mouse Model
Source: Pharmaceutics. 2025 Aug 12;17(8):1045. doi: 10.3390/pharmaceutics17081045 (PMC12389061; doi:10.3390/pharmaceutics17081045)
Supplement: Supplementary file 1 [file pharmaceutics-17-01045-s001.zip › pharmaceutics-3775088 Supplemental figure .pdf]

Korea Health Supplement Institute Agilent

**Sample name:**	D2024121390-1		
**Data file:**	2024-12-28 01-21-57+09-00D2024121390-1.dx	**Operator:**	Lee Ye Jin
**Instrument:**	KHFF-A-249	**Injection date:**	2024-12-28 01:22:55+09:00
**Inj. volume:**	10.000 µL	**Location:**	P1-C7
**Acq. method:**	Rosmarinic acid.amx	**Type:**	Sample
**Processing method:**	*3D UV Quantitative_DefaultMethod.pmx	**Calib Level:**	
		**Sample amount:**	0.00
**Manually modified:**	None		
**Processed Date**	2025-01-02 09:22:30+09:00		

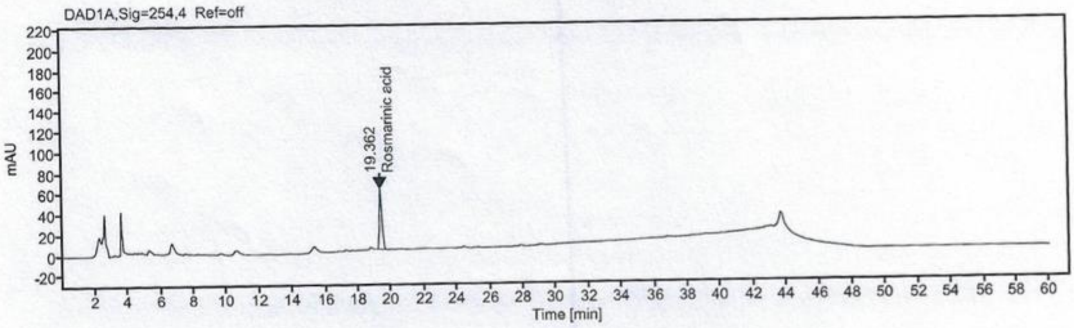

Signal: DAD1A,Sig=254,4 Ref=off

**Name**	**RT [min]**	**RF**	**Area**	**Peak Height**	**Concentration [µg/mL]**
Rosmarinic acid	19.36	12.523	664.410	59.187	53.056

Supplemental Figure S1. The content of rosmarinic acid in PE

Supplemental Figure 2.

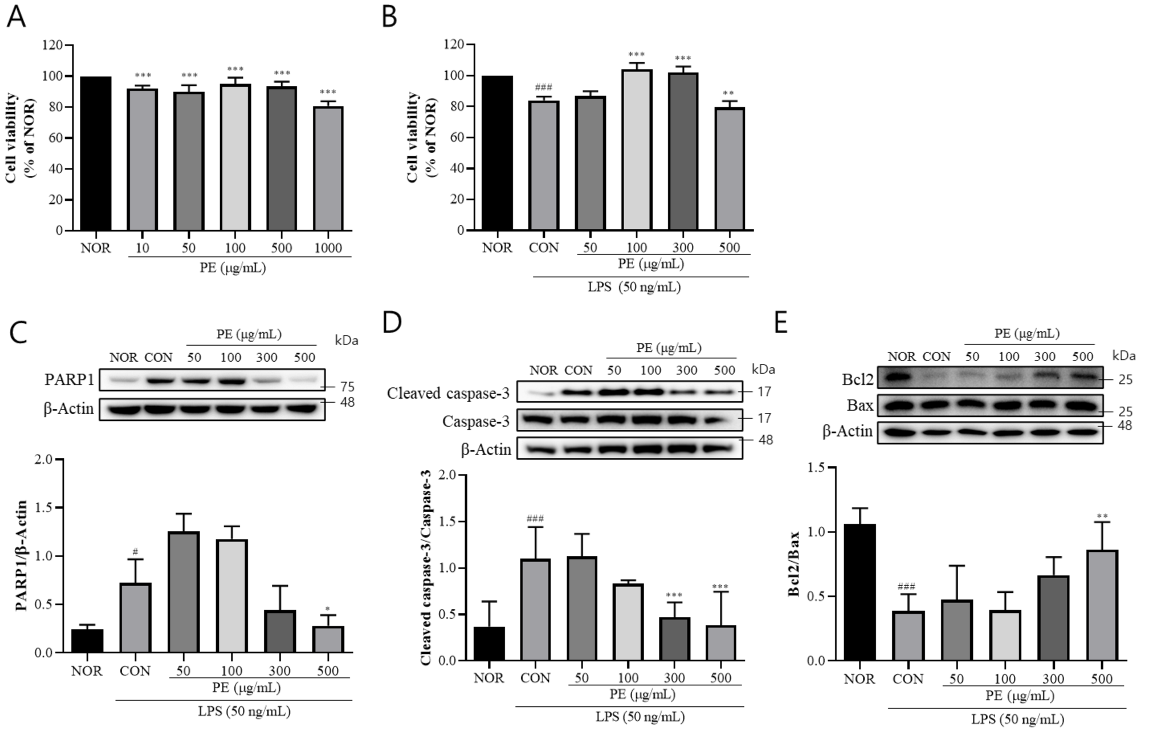

Supplemental figure 2. Effects of PE on LPS-induced cytotoxicity and apoptosis in BV2 microglial cells. (A) BV2 cells were treated with various concentrations of PE (10, 50, 100, 500, and 1000 μg/mL) for 24 h. Cell viability was determined using the MTS assay. (B) BV2 cells were pretreated with PE (50, 100, 300, and 500 μg/mL) for 2 h, followed by stimulation with LPS (50 ng/mL) for 24 h. Cell viability was measured by MTS assay. (C) Protein expression of PARP1 was analyzed by Western blotting after PE pretreatment and LPS stimulation. β-actin was used as a loading control. (E) The expression of Bcl-2 and Bax was evaluated by Western blot under the same treatment conditions, with β-actin as a loading control. Data are presented as mean ± SD. Statistical analysis was performed using one-way ANOVA followed by Newman-Keuls post hoc test. #P < 0.05, ###P < 0.001 vs. Normal (NOR) group; *P < 0.05, **P < 0.01, **P* < 0.05, ***P* < 0.01, ****P* < 0.001 vs. LPS-treated control (CON) group.
